# Plant growth-promoting rhizobacteria associated with avocado display antagonistic activity against *Phytophthora cinnamomi* through volatile emissions

**DOI:** 10.1371/journal.pone.0194665

**Published:** 2018-03-20

**Authors:** Alfonso Méndez-Bravo, Elvis Marian Cortazar-Murillo, Edgar Guevara-Avendaño, Oscar Ceballos-Luna, Benjamín Rodríguez-Haas, Ana L. Kiel-Martínez, Orlando Hernández-Cristóbal, José A. Guerrero-Analco, Frédérique Reverchon

**Affiliations:** 1 CONACYT—Escuela Nacional de Estudios Superiores, Laboratorio Nacional de Análisis y Síntesis Ecológica, Universidad Nacional Autónoma de México; Morelia, Michoacán, México; 2 Facultad de Ingenierías y Ciencias Químicas, Química Farmacéutica Biológica, Universidad Veracruzana; Xalapa, Veracruz, México; 3 Red de Estudios Moleculares Avanzados, Instituto de Ecología, A.C.; Xalapa, Veracruz, México; 4 Facultad de Biología, Universidad Veracruzana; Xalapa, Veracruz, México; 5 Escuela Nacional de Estudios Superiores, Laboratorio de Microscopía, Universidad Nacional Autónoma de México; Morelia, Michoacán, México; Karnatak University, INDIA

## Abstract

Rhizobacteria associated with crops constitute an important source of potentially beneficial microorganisms with plant growth promoting activity or antagonistic effects against phytopathogens. In this study, we evaluated the plant growth promoting activity of 11 bacterial isolates that were obtained from the rhizosphere of healthy avocado trees and from that of avocado trees having survived root rot infestations. Seven bacterial isolates, belonging to the genera *Bacillus*, *Pseudomonas* and *Arthrobacter*, promoted *in vitro* growth of *Arabidopsis thaliana*. These isolates were then tested for antagonistic activity against *Phytophthora cinnamomi*, in direct dual culture assays. Two of those rhizobacterial isolates, obtained from symptomatic-declining trees, displayed antagonistic activity. Isolate A8a, which is closely related to *Bacillus acidiceler*, was also able to inhibit *P*. *cinnamomi* growth *in vitro* by 76% through the production of volatile compounds. Solid phase microextraction (SPME) and analysis by gas chromatography coupled with mass spectrometry (GC-MS) allowed to tentatively identify the main volatiles emitted by isolate A8a as 2,3,5-trimethylpyrazine, 6,10-dimethyl-5,9-undecadien-2-one and 3-amino-1,3-oxazolidin-2-one. These volatile compounds have been reported to show antifungal activity when produced by other bacterial isolates. These results confirm the significance of rhizobacteria and suggest that these bacteria could be used for biocontrol of soil borne oomycetes through their volatiles emissions.

## Introduction

The ability of plants to adapt to different environments is largely conditioned by their associated microorganisms. The rhizosphere microbiota is particularly critical for plant development, health and productivity, nutrient cycling and other ecosystem processes [1]. The microbial communities present at the rhizosphere level actively interact with other organisms of the ecosystem in beneficial, harmful, or neutral ways. Such is the case for groups as abundant in the soil environment as actynomicetes; several genera of these gram-positive bacteria are able to grow associated with plant rhizosphere or as endophytes, and provide multiple benefits for plant fitness [2].

In agricultural systems, beneficial interactions may help counteract the loss of productivity due to soil erosion and impoverishment or to the occurrence of phytopathogens [3, 4]. Plant growth-promoting rhizobacteria (PGPR) may act positively on crop performance through direct or indirect mechanisms. Direct beneficial effects include solubilization of nutrients for plant absorption and the synthesis of diffusible or volatile phytoregulators [1, 5]. For example, some bacteria and fungi can enhance iron acquisition by the plant through the production of siderophores [2, 6] while other microorganisms can trigger induced systemic resistance [7]. Indirect effects on plant growth, on the other hand, include suppression of pathogens trough nutrient competition or through the production of antimicrobial compounds [8, 9, 10]. Rhizobacteria therefore constitute a source of potential biological control agents that could be used to develop sustainable alternatives to the application of synthetic agrochemical compounds.

Along with cereal production, avocado is the third most valuable agricultural commodity in Mexico, contributing to approximately 30% of the global production of avocado worldwide [11]. Despite the economic importance of avocado industry for Mexican agriculture, productivity of orchards has been hampered, among other reasons, by the high incidence of recalcitrant root system-associated pathogens. In avocado (*Persea americana* Mill.), Phytophthora root rot is caused by the oomycete *Phytophthora cinnamomi* Rand, a soil-borne pathogen which has been reported to affect more than 3,000 plant species and about 70% of avocado orchards [12]. *Phytophthora cinnamomi* attacks the feeder roots of avocado trees, which leads to root rot and consequently to branch-dieback, and is often followed by tree death. Studies aiming at isolating bacterial strains that are capable of inhibiting the growth of *P*. *cinnamomi* or that are associated with its suppressiveness have shown promising results and have identified *Bacillus* spp., *Pseudomonas* spp., *Streptomyces* spp. and some species of Actinobacteria as potential candidates for biological control of Phytophthora root rot [13, 14, 15, 16]. The antagonistic activity presented by rhizobacteria against fungal pathogens may occur through different mechanisms, such as the production of diffusible antifungal compounds or the emission of antifungal volatile compounds [17, 18]. Bacterial volatile compounds are particularly relevant in the search for *Phytopthora* biocontrol agents since they can reach further distances than bacterial diffusible compounds in the soil, and could enable interactions between physically separated microorganisms [19]. Previous reports have shown the antagonistic effects of volatiles produced by bacteria of the genera *Brevibacterium*, *Pseudomonas* and *Lysobacter* against *Phytophthora infestans* and *P*. *vignae* [20, 21, 22]. However, so far, the potential of bacterial volatile compounds for the suppression of *P*. *cinnamomi* has been poorly investigated.

Studying the culturable component of the avocado rhizobiome represents a promising strategy to identify beneficial microbial agents with direct or indirect effects on growth promotion, which could be integrated into alternative practices for pathogen management [23, 24]. The objectives of this study were therefore to: i) isolate and identify bacterial strains from the rhizosphere of Phytophthora root rot symptomatic and asymptomatic avocado trees, ii) evaluate their potential growth promoting effects on *Arabidopsis thaliana*, iii) assess their antagonistic activity against *P*. *cinnamomi*, the major root pathogen in Mexican avocado orchards, and finally iv) conduct a preliminary identification of the volatile compounds produced by the bacterial isolates with antifungal activity.

## Materials and methods

### Rhizosphere soil sampling and isolation of avocado rhizobacteria

**Fig 1:**
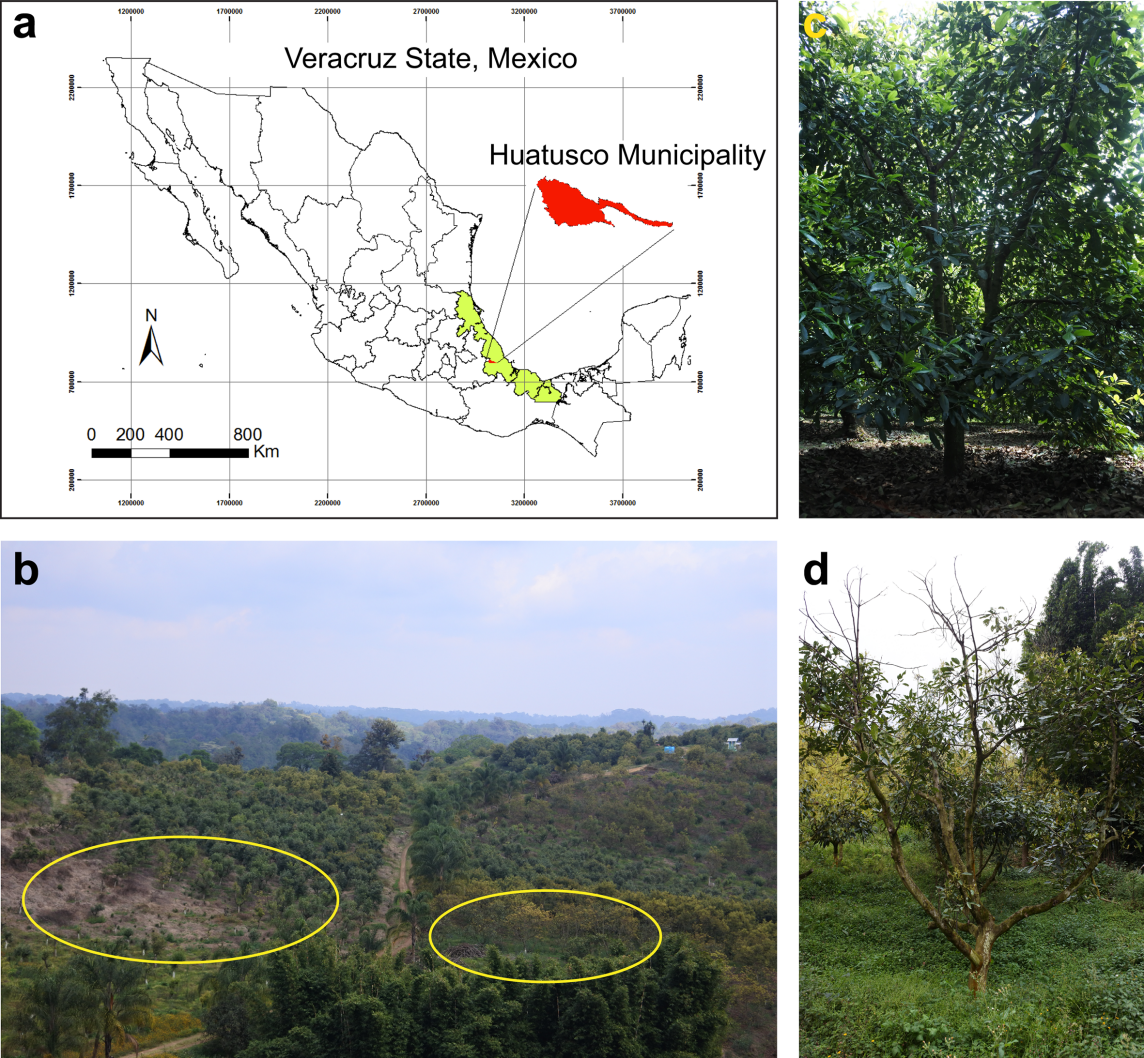
Localization and characteristics of sampling site. Geographic projection of Veracruz state and the Municipality of Huatusco (a); view of sampling zones (b), the symptomatic zone A is surrounded by yellow ovals; a representative healthy or symptomatic-declining tree is shown (c, d); map (a) was created using ArcMap 10.2.2. The authors generated digital information such as polygons and contours (public domain).

Sampling was carried out in August 2015 in an 8 year-old avocado orchard located in Huatusco, Veracruz State, in the Eastern side of Mexico (19° 10' 50" N and 96° 59' 59" W; Fig 1). Permission for sampling was awarded by the owner of the farm (Please see acknowledgments section). We selected ten avocado trees with symptoms of branch dieback in an area affected by root rot (zone A); root rot was confirmed by observing the youngest feeder roots. Additionally, ten asymptomatic avocado trees were selected from a root rot-free area (zone B) within the same orchard. Four soil and root samples were collected per tree, approximately 50 cm away from the trunk and at a depth of 15–30 cm. Samples were collected with a shovel which was washed with 70% ethanol between each sampling point, and transported at 4°C to the laboratory to be processed. Loose soil was removed from the roots, and the soil that was strongly adhered to the roots was recovered. One bulked sample per tree was obtained by mixing 1 g rhizosphere soil from each sampling point (n = 4). Subsequent dilutions were prepared from 1 g bulked rhizosphere soil (n = 10) and 99 ml sterile distilled water, and homogenized by shaking vigorously. Dilutions of 1:10 and 1:100 were then streaked onto Petri dishes with King Agar B medium (Cat. 60786; Sigma, St. Louis, MO), in triplicates. Plates were incubated at 24°C for three days. Bacterial isolates were taken from the plates as they grew and sub-cultured in LB agar until pure cultures were obtained. Culture purity was verified using standard Gram staining procedure and microscopic observation. Bacterial isolates from the same sampling zone were then grouped into morphotypes, based on criteria such as colonial form, color and texture, and cell shape, size and Gram staining results.

### Effect of rhizobacteria inoculation on *A*. *thaliana* development

*Arabidopsis thaliana* (Col-0 ecotype) seeds were surface-disinfected with 95% ethanol for 5 min and 20% sodium hypochlorite for 7 min. After five washes with sterile distilled water, seeds were stored at 4°C during 3 days for stratification and grown on agar plates containing 0.2X MS medium (Murashige and Skoog basal salts mixture, Cat. M5524; Sigma, St. Louis, MO). Plates were placed vertically at an angle of 65° to allow root growth along the agar surface in a plant-growth chamber with a photoperiod of 16 h of light, 8 h of darkness, light intensity of 200 μmol m^2^ s^-1^, and temperature of 23°C.

One bacterial isolate was randomly selected per morphotype and evaluated *in vitro* for its plant growth-promotion activity. Bacterial densities of 2.5 × 10^8^ CFU (Colony Forming Unit) were inoculated by streaking them on agar plates containing 0.2X MS medium. Seven day-old germinated *A*. *thaliana* seedlings (10 seedlings per plate) were transferred to control, non-inoculated media, or to one side of the plate, opposite to the bacterial inoculation site. Seedlings were placed at 2.5 cm (long distance) or 1 cm (close distance) of root tips from bacterial inoculum, to test the possibility that diffusible bacterial compounds could affect plant growth in a concentration-dependent manner. Plates were sealed with Parafilm® and were arranged in a completely randomized design into the plant-growth chamber. Experiments were performed in triplicate. Root system architecture and biomass accumulation were analyzed at 7 days after inoculation (dai).

Additionally, transgenic line *CycB1;1*:*uidA* [25] was employed to evaluate the effects of bacterial isolate A8a on primary root meristem activity by transferring 7-days old seedlings over bacterial inoculum. Seven days after germination, transgenic Arabidopsis seedlings were transferred to fresh MS 0.2X solid medium, placing root tips directly in touch with the A8a inoculum. Seven days after co-inoculation, expression of the *CycB1*:*uidA* marker was visually captured. For histochemical analysis of β-glucuronidase (GUS) activity, transgenic seedlings were incubated overnight at 37°C in a GUS reaction buffer [26]. Stained seedlings were cleared using the method of Malamy and Benfey [27]. At least 10 transgenic seedlings were analyzed per condition (non-inoculated control or inoculated seedlings). A representative seedling was chosen and photographed, using a Leica CME compound microscope.

### Molecular identification of rhizobacterial isolates with plant growth promoting effect

Bacterial isolates showing plant growth-promoting activity were identified through 16S rRNA gene sequencing. DNA was extracted from bacterial isolates using the DNeasy® Blood and Tissue kit (Qiagen, Germany), following the manufacturer’s instructions. The 16S rRNA region was amplified by PCR using universal primers 27F (5´-AGAGTTTGATCMTGGCTCAG-3´) and 1492R (5´-TACGGYTACCTTGTTACGACTT-3´), in 50 μl reactions containing 25–150 ng of template DNA, 1X of Taq buffer, 200 μM of each dNTP, 1.25 mM of MgCl_2_, 0.4 μM of each primer, and 0.5U of Taq DNA polymerase (Qiagen, Germany). Reactions were performed in a SureCycler 8800 (Agilent, California) under the following conditions: initial denaturation at 95°C for 4 min; 30 cycles of denaturation at 95°C for 45 s, annealing at 53°C for 45 s and extension at 72°C for 2 min; and a final extension step at 72°C for 5 min. Amplified DNA products were visually checked on an electrophoresis gel and purified using QiaQuick® Purification kit (Qiagen, Hilden, Germany), following the manufacturer's instructions. Purified DNA amplicons were then sent to Macrogen Inc. for sequencing. Sequences were deposited in GenBank (accession numbers MF686436 to MF686442).

Sequences were manually checked in BioEdit 7.2.5. [28] and aligned using MEGA 7 [29], using the multiple alignment program MUSCLE. The edited sequences and their best matches in GenBank nucleotide database (www.ncbi.nlm.nih.gov) were used to construct the alignment. A Maximum-Likelihood tree was constructed, using a Kimura two parameter model with Gamma distribution, and a Bootstrap method with 1000 replicates. Taxonomic assignations were corroborated with the ribosomal database project classifier tool [30].

### Antagonism of avocado rhizobacteria against *P*. *cinnamomi*

Bacterial isolates which promoted Arabidopsis growth were screened *in vitro* for antagonism activity against *P*. *cinnamomi*. Bacterial isolates were first re-streaked onto LB agar plates and incubated at 25°C during 48 h before setting up the dual culture assays. A culture of *P*. *cinnamomi* was grown on Potato Dextrose Agar (PDA) medium at room temperature for 5 days.

One plug of 5 mm of diameter was taken from the border of the *P*. *cinnamomi* mycelium and placed on the center of a PDA plate. Bacterial isolates were taken from a single colony with a sterile toothpick and inoculated at a 2 cm-distance from the mycelial plug, following the method recently reported [16]. Three different bacterial isolates were tested per plate and a sterilized toothpick mark was used as a control. All bacterial isolates were tested for antagonistic activity in triplicate. Dual culture assays were incubated for up to 11 days at room temperature. At day 5, mycelium radial growth was measured towards the bacterial treatment and the control, in order to calculate the percentage of inhibition of mycelial growth, using the following formula [31]:

%inhibition=[(R‑r)/R]×100,

where R is the radius of fungal growth from the center of the plate towards the control treatment, and r is the radius of fungal growth towards the bacterial treatment.

A bacterial isolate, which seemed to inhibit *P*. *cinnamomi* growth through volatile emissions (inhibition of mycelial growth observed in all growth directions), was further tested in dual plate experiments. Bacterial isolate A8a was inoculated in Petri dishes containing LB agar medium, in triplicate. A 5-mm mycelial plug of *P*. *cinnamomi*, taken from a seven days old culture plate, was placed in the center of another Petri dish containing PDA medium. Each bacteria-inoculated plate was placed upside of a PDA plate containing *P*. *cinnamomi*, sealed with Parafilm® and incubated at room temperature for seven days. The percentage of inhibition of fungal growth by bacterial volatiles was measured using the same formula as for the direct antagonism assays, with R as the radius of mycelial growth in the control treatment (*P*. *cinnamomi* only).

### Scanning electron microscopy (SEM) of *P*. *cinnamomi* mycelium

The hyphal morphology of *P*. *cinnamomi*, grown in indirect contact with isolate A8a for seven days, was observed and analyzed by scanning electron microscopy (SEM) in a JEOL JSM-IT300 microscope. Mycelial samples were fixed in a 4% glutaraldehyde solution for 24 h and washed with phosphate buffer (pH = 7.2). Mycelial samples were then dehydrated in a gradient of ethanol (70%, 80%, 90% and absolute ethanol). Samples were subsequently dried with liquid CO_2_ for 15 min in a Toussimis Autosamdri®-815, Series A, incorporated to aluminium stubs and sputter coated with 10-nm gold layer using Denton Desk V sputter coater. The scanning was performed with an accelerating voltage of 20 kV. Control mycelial samples, unexposed to isolate A8a, were processed similarly.

### Analysis of bacterial volatile emissions by solid phase microextraction (SPME) and gas chromatography-mass spectrometry (GC-MS)

The volatile compounds emitted by bacterial isolate A8a were analyzed following the procedure recently described [32], with some modifications. Briefly, bacterial isolate A8a was re-streaked onto LB agar plates in triplicate and incubated at room temperature for seven days. Volatiles were collected using solid-phase microextraction (SPME) fibers (Supelco, Bellefonte, PA with 50:30 μm divinylbenzene:carburen on polydimethylsiloxane [DVB/CAR/PDMS]). The fibers were inserted into the headspace of the LB agar plates during the whole incubation period. LB agar plates without bacteria were used as control and volatiles from the culture medium were captured under the same conditions. After seven days, volatiles were desorbed at 250°C for 10 min in a gas chromatographer (Perkin Elmer, Clarus 680) coupled to a mass analyzer (Perking Elmer, Clarus AQ8T). The desorption was carried out during 20 min and the operational conditions that were used were: initial oven temperature of 40°C for 3 min, increased to 160°C (15°C min^-1^), and further increased to 250°C (10°C min^-1^) with a total run time of 20 min. Helium gas was used as carrier gas (1.0 ml min^-1^, constant flow) and a Elite-5MS column (30 m, 0.25 mm I.D., 0.25 μm) was used as stationary phase. The mass spectrometer was operated in the electron ionization mode at 70 eV with a source temperature of 230°C, and with a continuous scan from 35 m/z to 500 m/z. The mass spectra, retention times, reverse and forward fit values (similarity index values) were compared with those reported in the NIST/EPA/NIH Mass Spectrometry Library of 2014 using Turbomass ver6.0.0 software (Perkin-Elmer Inc.). The volatile profile emitted by bacterial isolate A8a was contrasted with that emitted by the LB agar medium (control plates), and volatiles exclusively detected in isolate A8a were considered for preliminary identification according to the database of the NIST/EPA/NIH library. The relative abundances (%) of volatiles emitted by isolate A8a are expressed as the mean of three replicates ± standard deviation.

### Data analysis

Data analysis for the Arabidopsis-rhizobacteria assays was carried out from at least 30 seedlings. Arabidopsis development data were statistically analyzed using the SPSS 10 program, implementing multiple one-way ANOVA with a Tukey's post-hoc test to assess differences in growth and root developmental responses. All results were considered significant at *P* ≤ 0.05.

Data from antagonism assays were analyzed with the STATISTICA v.10 software. A one-way ANOVA followed by a Dunnett´s test was implemented to evaluate mycelial growth data in dual culture assays (direct inhibition). Mycelial growth inhibition by bacterial volatile compounds was analyzed with a Student's t-test.

## Results

### Isolation of rhizobacteria from symptomatic and asymptomatic avocado trees

In total, 46 bacterial isolates were obtained from the roots of symptomatic (zone A; Fig 1B, 1C and 1D) and asymptomatic (zone B) avocado trees. Bacterial isolates were named based on the sampling zone, number of sampled tree and a letter representing the isolate. Bacterial isolates were then grouped into 12 and 9 morphotypes for zone A and B respectively, based on their colonial and cellular morphological features. One bacterial isolate per morphotype (n = 21) was then selected for subsequent evaluation of plant growth promoting ability.

### Effects of inoculation with avocado associated rhizobacteria on growth of *A*. *thaliana*

**Fig 2:**
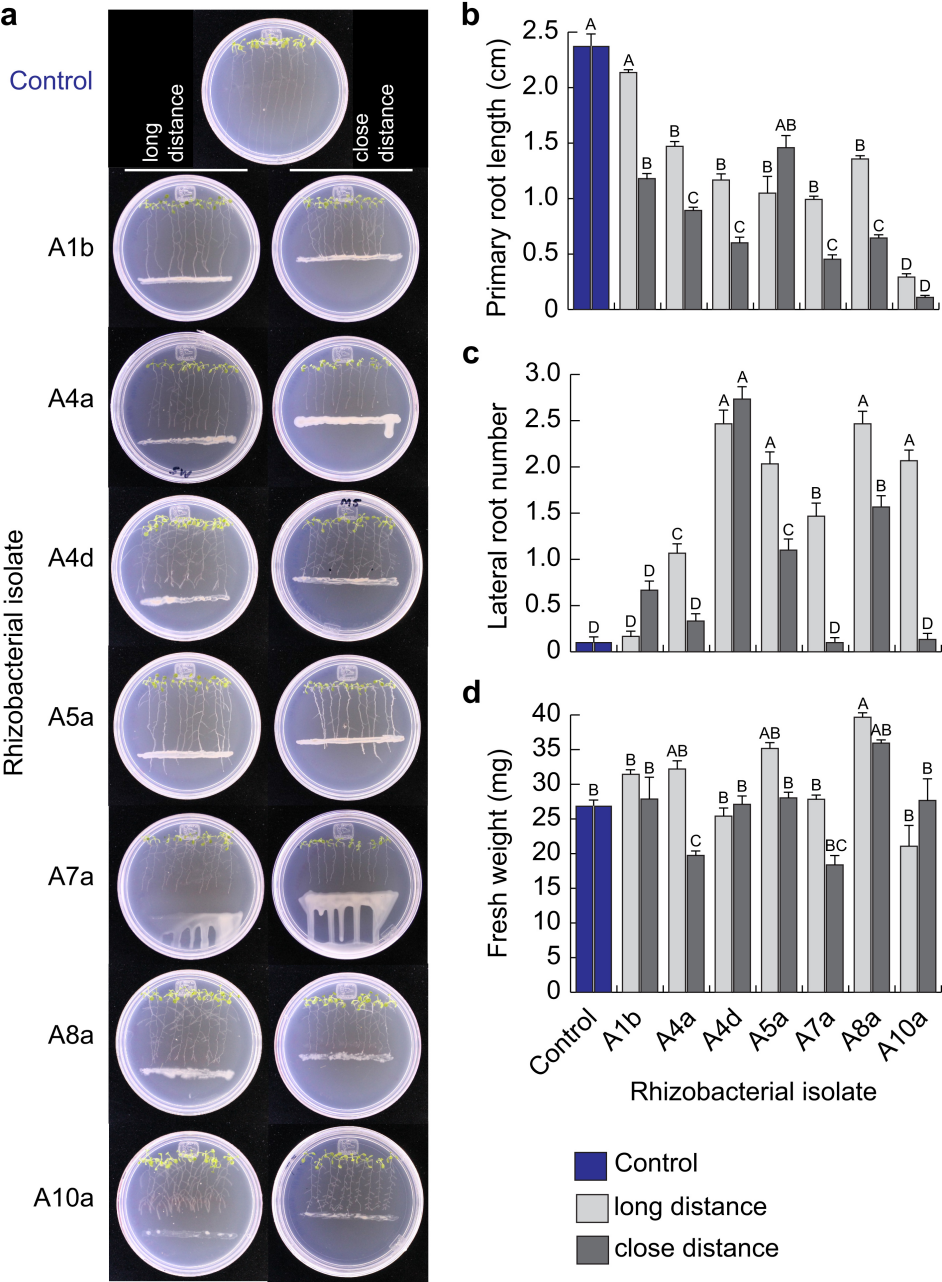
Co-cultivation of *Arabidopsis thaliana* seedlings with rhizobacterial isolates from symptomatic-declining avocado trees. Representative photographs of Arabidopsis Col-0 seedlings transferred to non-inoculated (Control) fresh media, or inoculated with rhizobacterial isolates at 2.5 cm (long distance) and 1 cm (close distance) from root tip. Primary root length (b), lateral root number (c) and fresh weight accumulation (d) were the developmental parameters analyzed. Data values represent the mean of 30 seedlings ± SE per treatment; different letters in graphs indicate significant differences (*P* < 0.05).

From the 21 selected rhizobacterial isolates, 11 were able to successfully grow on the plant growth culture medium MS (isolates A1b, A4a, A4d, A5a, A7a, A8a and A10a for zone A; and isolates B5b, B6a, B7a and B8a for zone B). Those 11 bacterial isolates were therefore selected for co-cultivation with Arabidopsis (Col-0 ecotype) seedlings *in vitro*, to determine their possible plant growth promoting activity. After 7 days of Arabidopsis co-cultivation with rhizobacteria, root system architecture and seedling fresh weight were evaluated. The effects of bacterial inoculation on Arabidopsis root growth were quite diverse, ranging from biostimulant (positive), deleterious (negative) or neutral (S1 Table). Distance from bacterial inoculum had a strong influence on the root development of Arabidopsis seedlings (Fig 2 and S1 Fig). With exception of bacterial isolate A1b, all tested isolates affected primary root growth by promoting lateral root formation (Fig 2A, 2B and 2C). When isolates A4d and A8a were inoculated at a distance of 1 cm (close distance) from root tip, stimulation of lateral root formation was increased (Fig 2A, 2B and 2C) as well as fresh weight accumulation (Fig 2D). Whilst long-distance inoculation of isolates A7a and A10a promoted lateral root development, closer inoculation affected the whole seedling development (Fig 2A, 2B, 2C and 2D).

**Fig 3:**
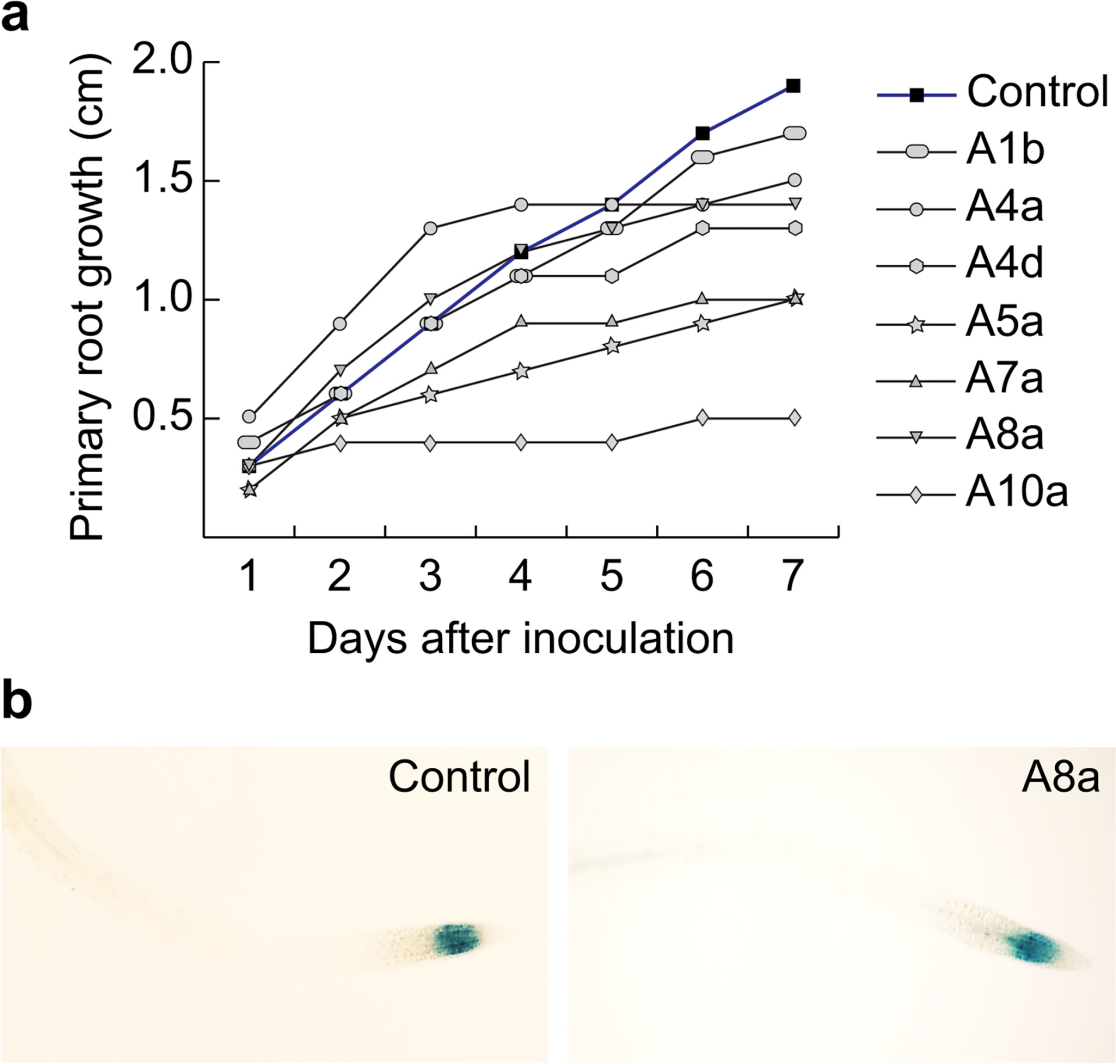
Effect of rhizobacterial isolates from symptomatic-declining avocado trees on *Arabidopsis thaliana* primary-root growth. Arabidopsis Col-0 seeds were germinated on agar plates containing MS 0.2X medium and transferred to control (uninoculated) or to rhizobacteria-inoculated fresh media at 2.5 cm from the root tip. (a) Length reached by primary root was measured during 7 days after inoculation (dai). (b) Expression of cell division marker *CycB1*:*uidA* primary-root meristem transferred to control or to A8a strain-inoculated medium during 7 days. Images were captured after histochemical β-glucuronidase assays. Mean ± SE values were plotted (*n* = 30).

To further detail alterations in primary root growth and promotion of developmental processes induced by rhizobacterial inoculation, growth kinetic assays were performed to compare early temporal effects of long distance inoculation on primary root length. Bacterial isolates A5a, A7a and A10a inhibited primary root growth on the second day after inoculation (2 dai; Fig 3A), compared with isolates with the highest growth promoting ability (A1b, A4a, A4d and A8a), which delayed primary root growth at 4 dai (Fig 3A). Rhizobacterial isolate A8a induced the strongest lateral root and fresh weight increase.

To elucidate whether primary root growth delay induced by strain A8a affected capacity for root cell division, we analyzed the expression of the cell-cycle marker *CycB1*:*uidA* [25] in Arabidopsis transgenic seedlings. At seventh day after germination, transgenic seedlings were transferred to fresh MS 0.2X solid medium, placing root tips directly in touch with the A8a inoculum. At 7 days after inoculation (dai), expression of the *CycB1*:*uidA* marker was visually captured. Histochemical assays showed that GUS activity in meristematic region of A8a-inoculated roots was not altered by bacterial growth, when compared with that of non-inoculated (control) roots (Fig 3B). The results suggest that plant growth-promoting activity of the A8a strain does not compromise root cell division capacity.

### Identification of plant growth-promoting rhizobacteria

**Fig 4:**
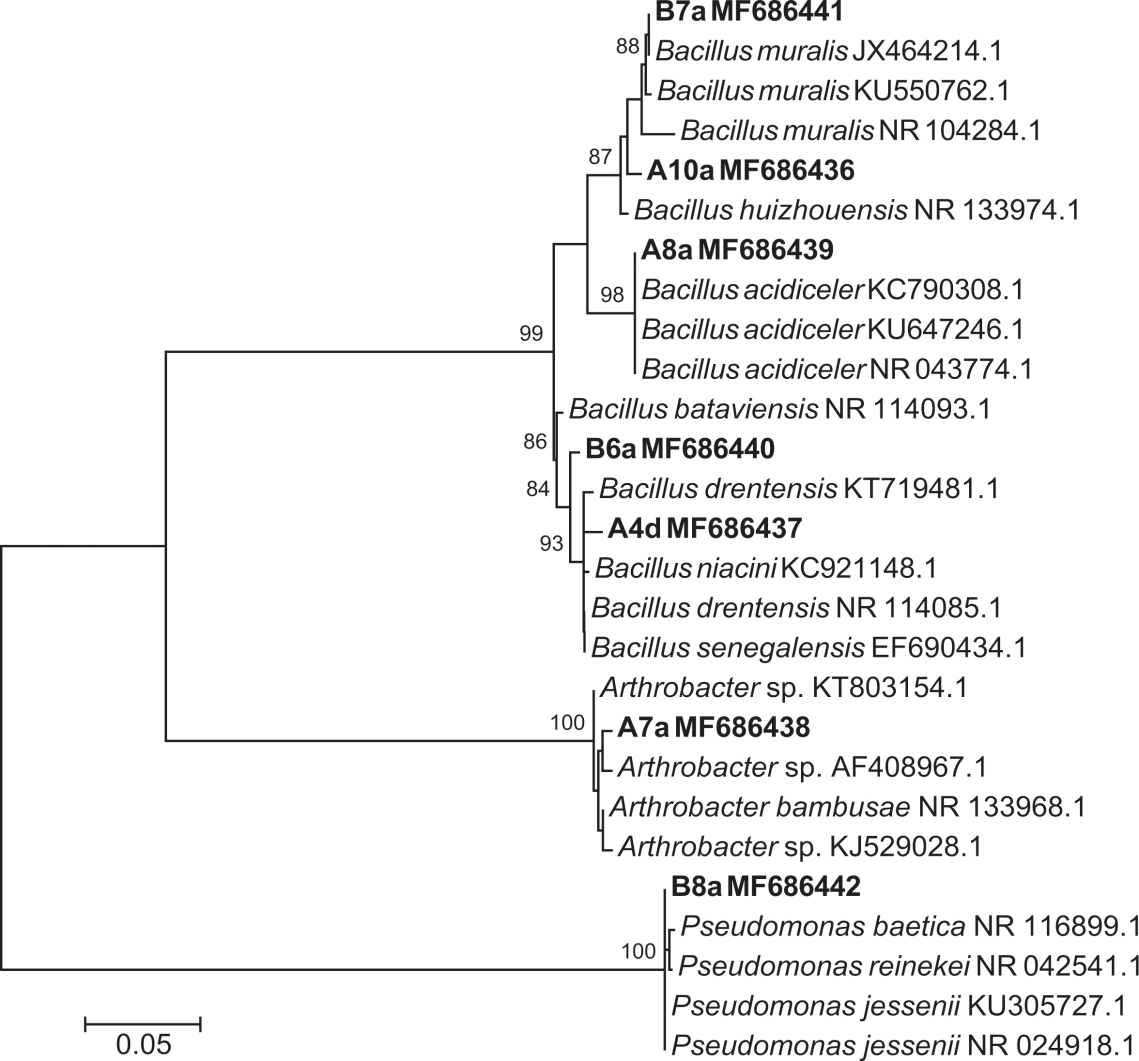
Maximum-Likelihood tree of partially sequenced 16S rRNA genes. Bold letters indicate rhizobacterial isolates that were obtained in this study. Values above nodes are bootstrap values obtained from 1000 replicates.

Bacterial isolates showing plant growth-promoting activity were identified through 16S rRNA gene sequencing. Sequence closest matches, based on BLAST similarity analysis, are presented in Table 1. Most sequenced isolates belonged to the *Bacillus* genus, despite the initial culturing of bacterial isolates on the Gram negative-semi selective medium King Agar B. The Maximum-Likelihood tree presented in Fig 4 shows that five of the seven growth-promoting bacterial isolates belonged to the genus *Bacillus*, and were closely related to *B*. *muralis*, *B*. *huizhouensis*, *B*. *acidiceler*, *B*. *bataviansis* and *B*. *drentensis*. The other two isolates corresponded to the genus *Arthrobacter* (A7a isolate) and *Pseudomonas* (B8a isolate), respectively (Fig 4).

**Table 1:** Molecular identification of avocado rhizobacteria with plant growth promoting activity by 16S rRNA sequencing. Taxonomic assignment was determined with the rdp classifier tool. Sequence closest matches were based on the NCBI database “16S ribosomal RNA sequences (Bacteria and Archaea)”.

ID rhizobacterial strain	GenBank accession number	Taxonomic assignment	NCBI best match (accession number)	Identity (%)
A4d	MF686437	*Bacillus* sp.	*Bacillus drentensis* (NR_114085.1)	99
A7a	MF686438	*Arthrobacter* sp.	*Arthrobacter bambusae* (NR_133968.1)	99
A8a	MF686439	*Bacillus* sp.	*Bacillus acidiceler* (NR_043774.1)	99
A10a	MF686436	*Bacillus* sp.	*Bacillus huizhouensis* (NR_133974.1)	98
B6a	MF686440	*Bacillus* sp.	*Bacillus bataviensis* (NR_114093.1)	98
B7a	MF686441	*Bacillus* sp.	*Bacillus muralis* (NR_042083.1)	99
B8a	MF686442	*Pseudomonas* sp.	*Pseudomonas baetica* (NR_116899.1)	99

### Antagonistic effects of plant growth promoting rhizobacteria on *P*. *cinnamomi in vitro*

**Fig 5:**
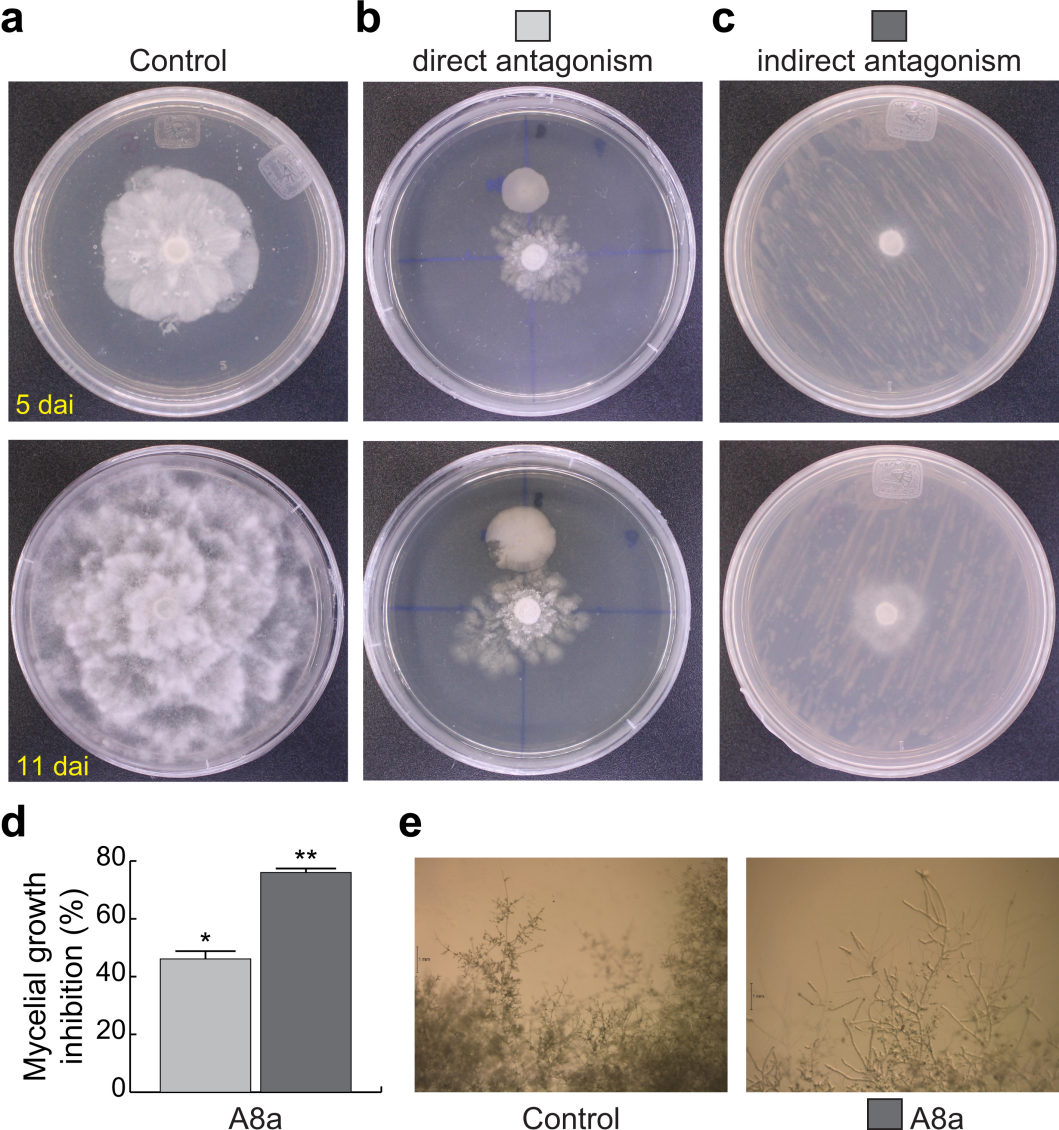
Antagonical activity of isolate A8a, phylogenetically close to *Bacillus acidiceler*, against *Phytophthora cinnamomi*. Disks of *P*. *cinnamomi* mycelium were grown on agar PDA-containing plates. Radial growth was monitored for 11 days in non-inoculated conditions (a). Confrontation with *B*. *acidiceler* was performed by directly co-cultivating mycelial disks with bacterial inoculum, at a distance of 2 cm (b), or indirectly, growing them on the opposite halves of the same Petri dish (c). Assays were carried out by triplicate. Representative plates were photographed at day 5 and 11 after inoculation (dai). At 7 dai, the inhibition percentage of mycelial radial growth by direct (light gray bars) or indirect (dark gray bars) antagonism was analyzed (d); hyphal deformations induced by indirect inoculation were visually analyzed by stereoscopic observations at 7 dai (e). Values shown in (d) represent the mean of three replicates ± SD; asterisks indicate significant inhibition (*P* ≤ 0.05).

In order to explore the potential of our rhizobacterial isolates to antagonize growth and development of some soil borne pathogens, we evaluated the ability of our seven identified plant growth-promoting isolates to inhibit *in vitro* the mycelial growth of *P*. *cinnamomi* in dual culture assays. Only isolates A4d and A8a, both belonging to the genus *Bacillus*, inhibited mycelial growth after 5 days in direct confrontation with the phytopathogen (Table 2). Isolate A8a, determined to be phylogenetically close to *B*. *acidiceler*, presented a strong antagonistic activity against *P*. *cinnamomi*, inhibiting the oomycete mycelial growth for until 11 dai (Fig 5A and 5B). The radial growth of mycelium was inhibited by 46% at 7 dai (Fig 5D).

**Table 2:** Direct antagonism assays of avocado rhizobacteria against *Phytophthora cinnamomi*. Mycelial growth was measured at fifth day after dual culture.

ID rhizobacterial strain	Taxon (NCBI best match)	Mycelial growth mm ± SD	Mycelial growth inhibition (%) ^†^
A4d	*Bacillus drentensis*	21.67 ± 3.06	14.5
A7a	*Arthrobacter bambusae*	25.33 ± 2.08	n
A8a	*Bacillus acidiceler*	13.67 ± 2.04	46.1
A10a	*Bacillus huizhouensis*	21.67 ± 2.08	n
B6a	*Bacillus bataviensis*	24.33 ± 2.52	n
B7a	*Bacillus muralis*	23.67 ± 5.51	n
B8a	*Pseudomonas baetica*	19.67 ± 2.52	n

^†^n means inhibition less than 10%.

Values represent average of 3 replicates; percentage of growth inhibition was calculated respect to mycelial growth in control conditions (24.00 ± 1.53 cm).

### Antifungal activity of volatiles emitted by isolate A8a on *P*. *cinnamomi*

**Fig 6:**
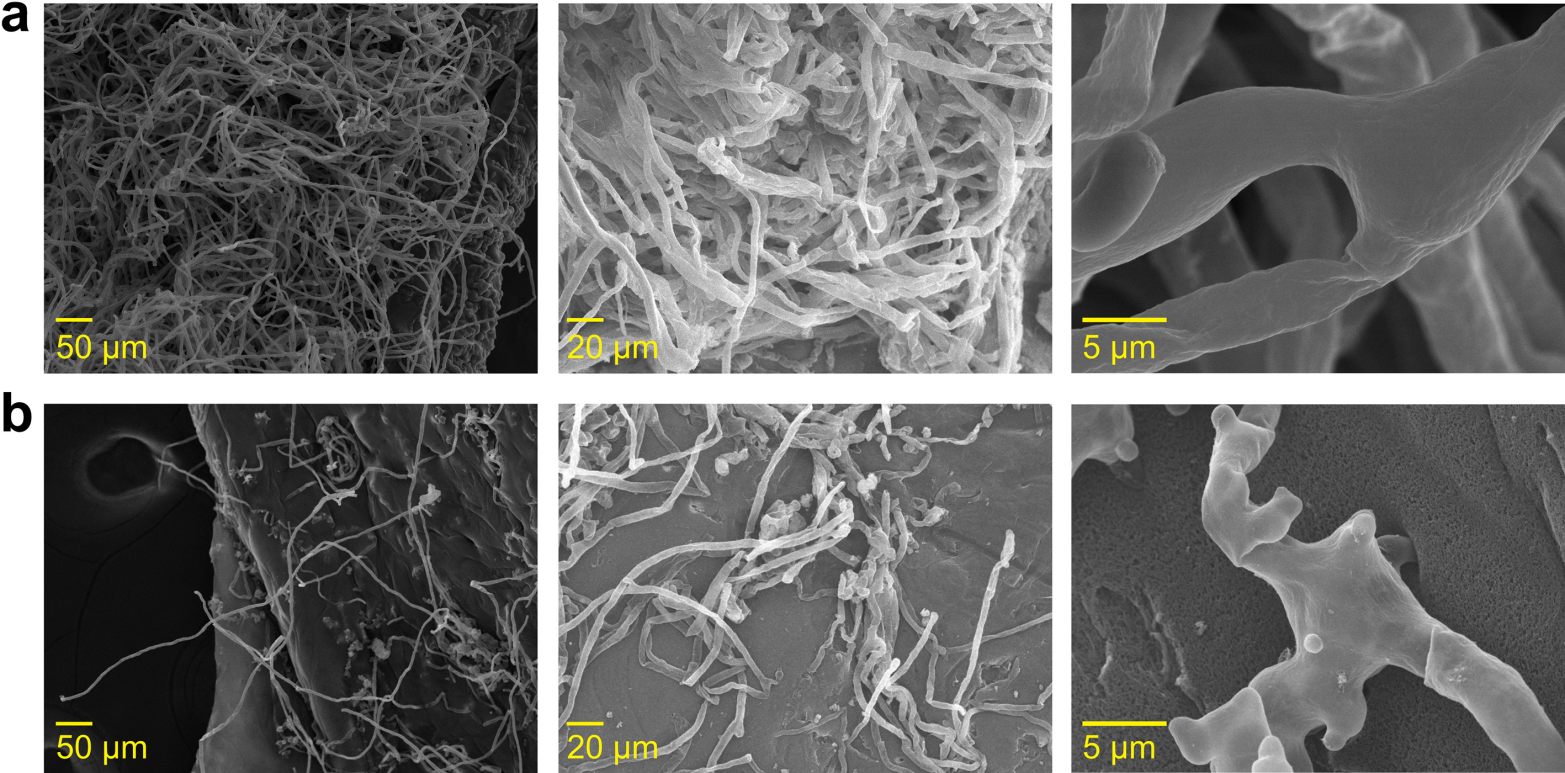
Scanning electron microscopy of *P*. *cinnamomi* mycelium antagonized by volatiles emitted by isolate A8a, phylogenetically close to *Bacillus acidiceler*. After 7 d, control mycelium (a) was compared with mycelium indirectly exposed to bacteria-inoculated plates (b).

We evaluated the antifungal activity of volatile compounds emitted by isolate A8a against *P*. *cinnamomi*, by using the two-sealed-base-plates method, avoiding direct contact between the bacteria and the oomycete. Radial growth of mycelium was measured at 5 and 11 dai (Fig 5C). The antagonistic effect of bacterial volatiles was stronger than the effect observed when isolate A8a and *P*. *cinnamomi* were cultured in direct interaction at 7 dai (Fig 5D), reaching up to 76% mycelium growth inhibition; alterations on hyphal and conidiophore morphology were visually registered at 7 dai by stereoscopic observations (Fig 5E). Additionally, a deeper analysis of changes in mycelium morphology induced by volatiles emitted by isolate A8a was conducted at 7 dai with scanning electron microscopy (SEM). The SEM micrographs of the mycelium of *P*. *cinnamomi*, being exposed to bacterial volatiles, clearly indicate that multiple degenerative alterations occur in the hyphal morphology, when compared with the control, non-exposed mycelium (Fig 6A and 6B). Shriveled hyphal walls and lower hyphal density were observed in the antagonized mycelium.

### Analysis of volatiles emitted by isolate A8a

Because of the strong antagonistic activity on *P*. *cinnamomi* growth exhibited by the volatiles emitted by bacterial isolate A8a, we analyzed the volatile profile of this *Bacillus* species by SPME-GC-MS. The total ion chromatogram (S2 Fig) of isolate A8a displayed a total of seven major components (Table 3), which were identified to belong to the chemical categories of ketones, aldehydes, alkyls, sulfoxides, pyrazines and alcohols, respectively. On the basis of retention times, m/z ratios and mass index values (forward fit and reverse fit values), the three most abundant volatiles were tentatively identified as 2,3,5-trimethylpyrazine, 3-amino-1,3-oxazolidin-2-one and 6,10-dimethyl-5,9-undecadien-2-one, with relative abundances of 28.86%, 22.80% and 15.95%, respectively.

**Table 3:** Tentative identification of volatiles emitted by bacterial isolate A8a through SPME and GC-MS^†^.

Compound ID	Retention time (min)	Relative abundance (%)	Chemical category	Reverse fit and Forward fit values (RF:FF)
3-amino-1,3-oxazolidin-2-one	2.88 ± 0.02	15.95 ± 2.56	Ketone	783:738
(5E,9E)-6,10-Dimethyl-5,9-dodecadien-2-one	3.06 ± 0.02	7.18 ± 1.94	Ketone	902:839
3-methylpentanal	3.33 ± 0.02	2.25 ± 0.49	Aldehyde	875:816
(methyldisulfanyl)methane	3.94 ± 0.01	8.88 ± 1.49	Sulfoxide	965:924
2,3,5-Trimethylpyrazine	8.17 ± 0.03	28.86 ± 8.24	Pyrazine	947:866
2-Phenylethanol	9.56 ± 0.04	14.08 ± 1.55	Alcohol	954:886
6,10-dimethyl-5,9-undecadien-2-one	12.82 ± 0.08	22.80 ± 10.87	Ketone	860:702

^†^Reported compounds include those emitted by bacterial isolate A8a but not emitted by the control plates.

Data are expressed as means ± SD of three replicates. The tentative names of volatile compounds were given according to the information provided by the NIST/EPA/NIH Mass Spectrometry Library 2014. Fit values were obtained using TurboMass Software and the maximum obtainable value is 1,000, which represents a perfect match between the search spectrum and the library entry.

## Discussion

Microbes colonizing the rhizosphere may interact with plants in a beneficial manner, by suppressing plant diseases through the production of antagonistic compounds or by promoting plant growth and defense responses [33]. The integration of beneficial plant-microbe and microbe-phytopathogen interactions can potentially offer new strategies to improve plant productivity in an environmentally friendly manner [34]. Previous studies have shown that upon attack by soil-borne pathogens, plants can exploit microbial consortia from soil for protection against infections, restructuring bacterial communities associated with their rhizosphere [8]. Rhizobacteria retrieved from healthy trees may also differ from those associated with infected trees because they play a role in disease suppression, or merely because they reflect changes in root exudate patterns due to the infection [23].

In this study, we identified several PGPR, which were isolated from the avocado rhizosphere of symptomatic-declining or healthy trees (Fig 1). Although most tested isolates inhibited the primary root elongation of *A*. *thaliana* seedlings (Fig 2 and S1 Fig), only seven isolates significantly promoted the formation of lateral roots, a previously described root architectural trait induced by the PGPR *Pseudomonas fluorescens* WCS417 and *Bacillus amyloliquefaciens* subsp. *plantarum* UCMB5113 in interaction with Arabidopsis [35, 36]. These PGPR belonged to the bacterial genera *Bacillus*, *Pseudomonas* and *Arthrobacter* (Table 1 and Fig 4). Species of *Bacillus* and *Pseudomonas* have been widely documented to enhance plant growth in several studies, through their siderophore production [37, 38], their phytohormone production or signaling [35, 39, 40], their ability to solubilize phosphate and fix nitrogen [41] or their antimicrobial activities [42, 43]. Although less studied, *Arthrobacter* species also present plant growth promoting activity, as previously reported [44]: *Arthrobacter* sp., isolated from the tomato rhizosphere, showed high phosphate solubilizing ability and indole-acetic acid (IAA) production.

Interestingly, the root architectural remodeling effect induced by rhizobacterial isolate A8a seemed to be a retardant, more than an inhibitor of primary root growth, as evidenced by the growth kinetic analyses (Fig 3A), by the activity of the cell division marker CycB1:uidA at the meristematic region (Fig 3B) and by the promotion of biomass accumulation (Fig 2D). Future studies should be directed at elucidating the endogenous signals that modulate plant developmental changes induced by the rhizobacterial isolates described in this work.

Rhizobacteria have proved to be a source of biological control agents of fungal phytopathogens in numerous studies [16, 31, 43, 45]. In the present work, we found that isolate A8a, a phylogenetically close relative to *Bacillus acidiceler*, presented strong antagonism against *P*. *cinnamomi*, through the emission of both diffusible and volatile compounds (Table 2, Fig 5 and Fig 6). Species of the bacterial genus *Bacillus* have shown to inhibit the mycelial growth of several fungal pathogens, such as *Fusarium euwallaceae* [16, 46], *F*. *oxysporum* and *Rosellinia necatrix* [15] and *Phytophthora* spp. [16, 47]. Fungal growth inhibition by *Bacillus* spp. has been reported to occur through different mechanisms. *Bacillus subtilis* and *B*. *amyloliquefaciens* can produce different lipopeptides which antifungal activity varies according to the targeted phytopathogen [48, 49]. For example, iturin, a lipopeptide synthesized by *B*. *amyloliquefaciens*, is able to inhibit the growth of *Alternaria panax*, *Botrytis cinerea*, *Colletotrichum orbiculare*, and *Penicillium digitatum*, among other fungal pathogens [50]. On the other hand, fengycin, another lipopeptide synthesized by *B*. *amyloliquefaciens*, presents antifungal activity against *F*. *oxysporum*, *F*. *solani*, *Verticillium dahlia* and *Phytophthora parasitica* [48]. The lipopeptides produced by *B*. *acidiceler*, the closest relative species of strain A8a, are still unknown and further work is therefore aimed at unravelling the chemical composition of antifungal substances secreted by strain A8a.

*Bacillus* species are also able to emit volatile organic compounds with antifungal properties [51, 52]. Those volatiles include benzene compounds, pentadecane, tetradecane, and some ketones, and are of special interest due to their long distance action [52, 53]. In this study, the main volatile compounds produced by isolate A8a were ketones, aldehydes, alkyls, sulfoxides, pyrazines and alcohols, which were able to inhibit *P*. *cinnamomi* mycelial growth by 76% (Fig 5C and 5D). Some of the most abundant volatile compounds tentatively identified in our analysis have previously been reported for their antifungal properties. For instance, (methyldisulfanyl)methane (IUPAC name; Table 3), reported previously as dimethyl disulfide and produced by *Bacillus cereus* C1L, showed antagonistic effects against *Botrytis cinerea* [54]. Dimethyl disulfide and other sulfur-containing compounds emitted by *Pseudomonas* species were recently shown to stop the growth of *Phytophthora infestans* and to present sporicidal activity [55]. Other volatile compounds produced by *Pseudomonas* strains, such as 1-Undecene, are also able to reduce sporangia formation and the release of zoospores in *P*. *infestans* [21]. The volatile compound 2-phenylethanol (Table 3) inhibited mycelial growth of *Penicillium digitatum*, *P*. *italicum* and *B*. *cinerea* [56, 57]. Additionally, the 2,3,5-trimethylpyrazine compound has been identified as an abundant component in several volatile profiles from fungistatic soils against *Rhizoctonia* and *Fusarium* phytopathogens [58]. Pyrazine compounds produced by *Lysobacter*, *Pseudomonas* and *Bacillus* species have also been reported to inhibit the growth of *Phythophthora infestans* and *P*. *capsici* [55, 59, 22].

Bacteria belonging to the genus *Bacillus* have been considered to be good candidates from which to develop biologically active formulations, due to their ability to produce endospores that favor long-term storage and to their environmental ubiquity [60]. Further studies should therefore be aimed at evaluating *in vivo* the potential of volatile and diffusible compounds emitted by bacterial isolate A8a, in order to verify their potential use for avocado protection in experimental settings that are closer to field conditions.

## Conclusions

In this study, we characterized the growth promoting activity of 7 bacterial isolates that were obtained from the rhizosphere of healthy and symptomatic-declining avocado trees. These isolates belonged to the genera *Bacillus*, *Pseudomonas* and *Arthrobacter*. Isolates A4d and A8a were also able to inhibit the growth of *Phytophthora cinnamomi* in direct interactions-assays. Isolate A8a, which is closely related to *Bacillus acidiceler*, produced volatile compounds that reduced *P*. *cinnamomi* mycelial growth by 76%, and were preliminary identified by SPME-GC-MS as 2,3,5-trimethylpyrazine, 6,10-dimethyl-5,9-undecadien-2-one and 3-amino-1,3-oxazolidin-2-one. These results confirm the significance of rhizobacteria and bacterial volatile compounds for the control of soil borne oomycetes and the potential of *Bacillus* species for the development of biologically active formulations, such as biofertilizers or biofungicides.

## Supporting information

S1 Table Bacterial isolates obtained from avocado rhizosphere and effect on *Arabidopsis thaliana* seedlings. ^†^The effect of rhizobacteria isolated from avocado symptomatic trees (A) or healthy trees (B) on Arabidopsis seedlings *in vitro* at seventh day after inoculation. The effects were divided into three categories; Positive, visible growth promotion effect compared to control non inoculated; Negative, visible damage effect compared to control; Neutral, no visible positive or negative effect compared to control non inoculated seedlings.
(DOCX)

S1 Fig Co-cultivation of *Arabidopsis thaliana* seedlings with rhizobacterial isolates from healthy non-symptomatic avocado trees. Representative photographs of Arabidopsis Col-0 seedlings inoculated with rhizobacterial isolates at 2.5 cm (long distance) and 1 cm (close distance) from root tip. Primary root length (b), lateral root number (c) and fresh weight accumulation (d) were the developmental parameters analyzed. Data values represent one of three independent plates that gave similar results, 10 seedlings were employed per treatment.
(TIF)

S2 Fig Representative total ion chromatogram of volatile emissions by PGPR isolate A8a. The volatile profile of the control sample (LB agar medium; a) was contrasted with the profile of volatiles emitted by isolate A8a (b). Arrows indicate differential peaks of compounds described in Table 3.
(TIF)
