# Supplementary material for: Plant growth-promoting rhizobacteria associated with avocado display antagonistic activity against Phytophthora cinnamomi through volatile emissions
Source: PLoS One. 2018 Mar 20;13(3):e0194665. doi: 10.1371/journal.pone.0194665 (PMC5860777; doi:10.1371/journal.pone.0194665)
Supplement: S1 Table — †The effect of rhizobacteria isolated from avocado symptomatic trees (A) or healthy trees (B) on Arabidopsis seedlings in vitro at seventh day after inoculation. The effects were divided into three categories; Positive, visible growth promotion effect compared to control non inoculated; Negative, visible damage effect compared to control; Neutral, no visible positive or negative effect compared to control non inoculated seedlings. (DOCX) [file pone.0194665.s001.docx]

**Supporting information**

**S1 Table.** Bacterial isolates obtained from avocado rhizosphere and effect on *Arabidopsis thaliana* seedlings

| Isolate number | ID rhizobacterial isolate | Effect *in vitro* on Arabidopsis development^†^ | | |
| --- | --- | --- | --- | --- |
|  |  | Long distance | | Close distance |
| 1 | A1b | positive | positive | |
| 2 | A4a | positive | neutral | |
| 3 | A4d | positive | positive | |
| 4 | A5a | positive | neutral | |
| 5 | A7a | negative | negative | |
| 6 | A8a | positive | positive | |
| 7 | A10a | positive | neutral | |
| 8 | B5b | neutral | neutral | |
| 9 | B6a | positive | neutral | |
| 10 | B7a | negative | negative | |
| 11 | B8a | positive | positive | |

^†^The effect of rhizobacteria isolated from avocado symptomatic trees (A) or healthy trees (B) on Arabidopsis seedlings *in vitro* at seventh day after inoculation. The effects were divided into three categories; Positive, visible growth promotion effect compared to control non inoculated; Negative, visible damage effect compared to control; Neutral, no visible positive or negative effect compared to control non inoculated seedlings.
