# Supplementary figures and images for: Plant growth-promoting rhizobacteria associated with avocado display antagonistic activity against Phytophthora cinnamomi through volatile emissions
Source: PLoS One. 2018 Mar 20;13(3):e0194665. doi: 10.1371/journal.pone.0194665 (PMC5860777; doi:10.1371/journal.pone.0194665)

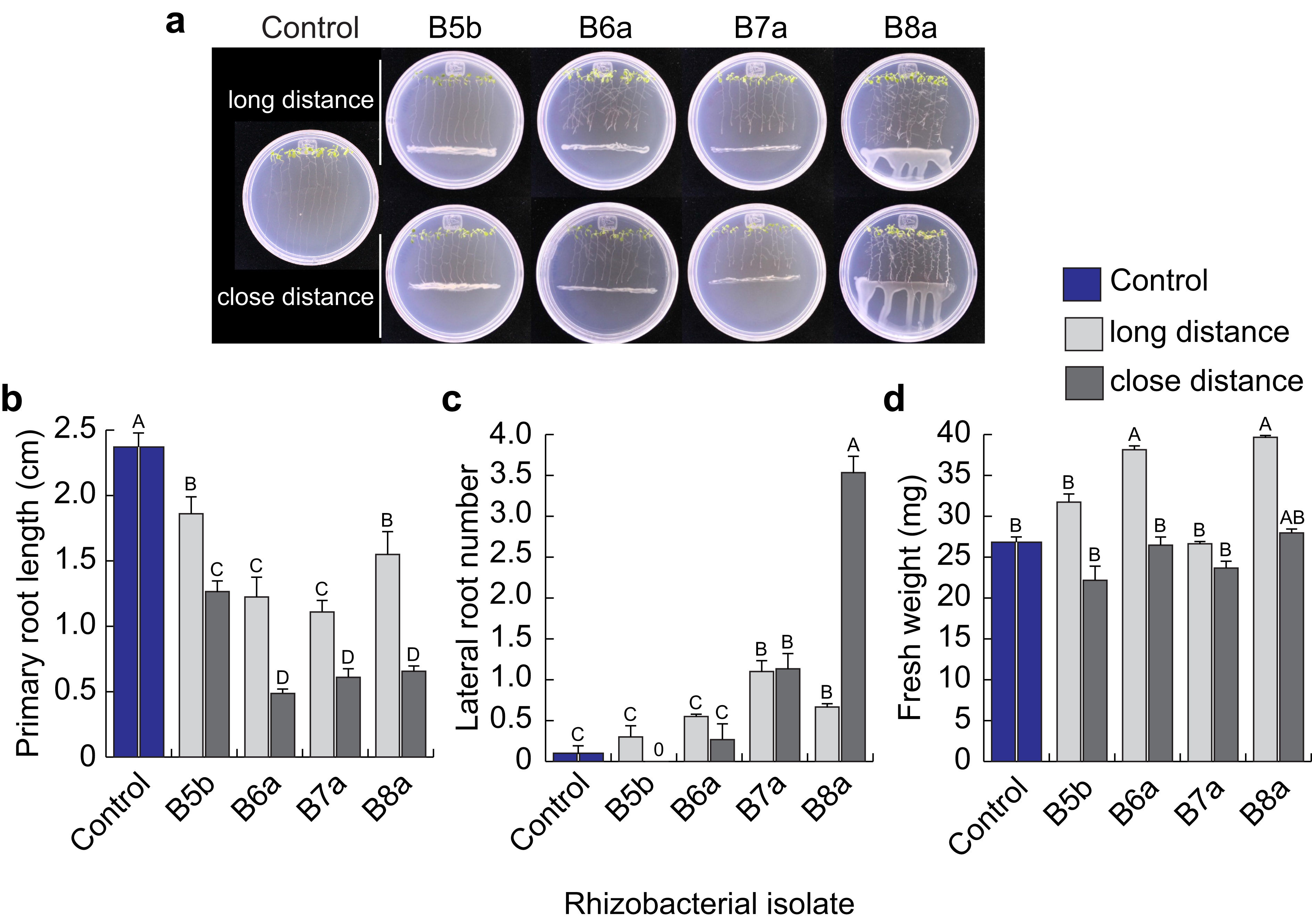

Supplement: S1 Fig — Representative photographs of Arabidopsis Col-0 seedlings inoculated with rhizobacterial isolates at 2.5 cm (long distance) and 1 cm (close distance) from root tip. Primary root length (b), lateral root number (c) and fresh weight accumulation (d) were the developmental parameters analyzed. Data values represent one of three independent plates that gave similar results, 10 seedlings were employed per treatment. (TIF) [file pone.0194665.s002.tif]

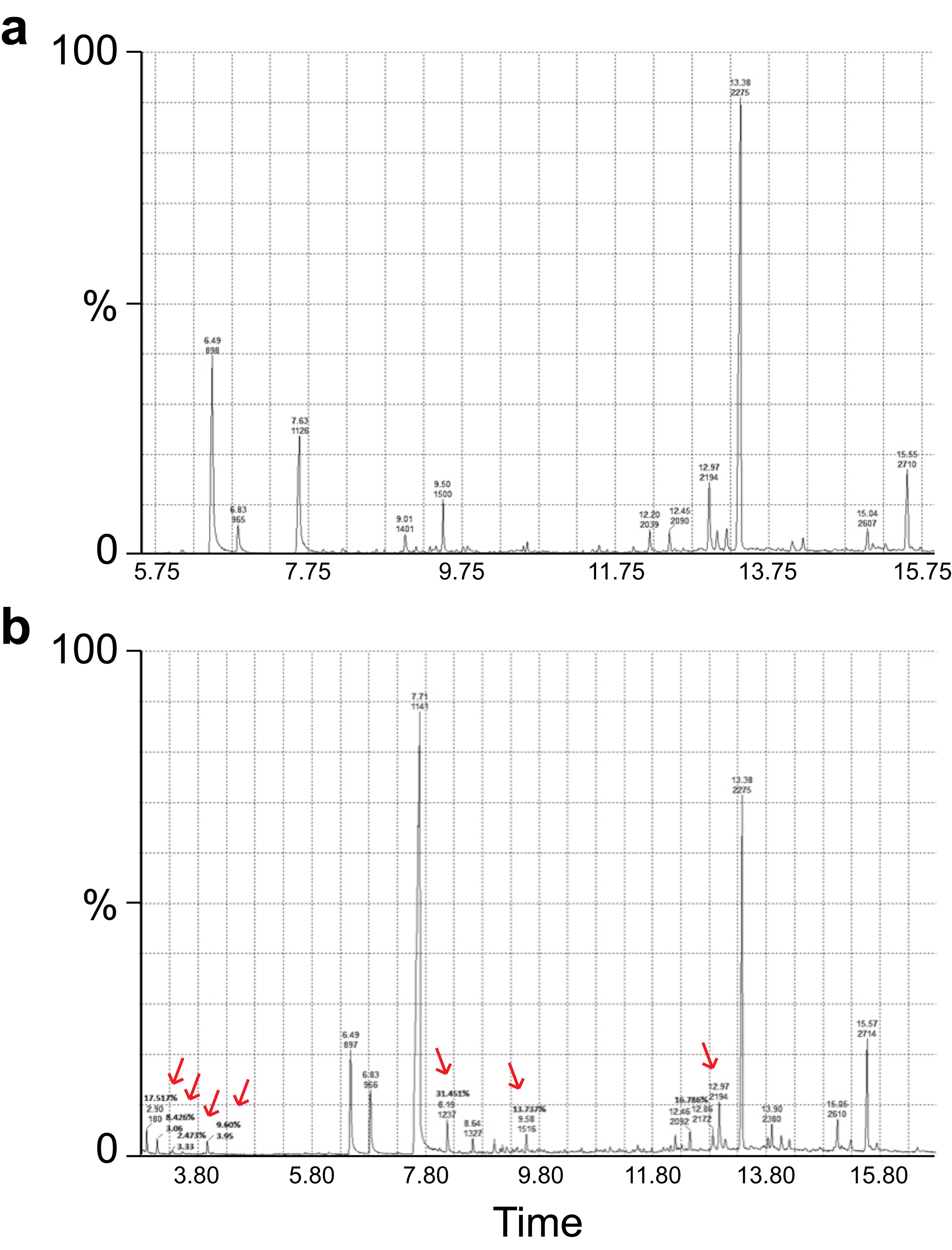

Supplement: S2 Fig — The volatile profile of the control sample (LB agar medium; a) was contrasted with the profile of volatiles emitted by isolate A8a (b). Arrows indicate differential peaks of compounds described in Table 3. (TIF) [file pone.0194665.s003.tif]
